# Dihydropyrimidine dehydrogenase deficiency and fluorouracil-related toxicity

**DOI:** 10.1038/sj.bjc.6690098

**Published:** 1999-02

**Authors:** G Milano, M C Etienne, V Pierrefite, M Barberi-Heyob, R Deporte-Fety, N Renée

**Affiliations:** Oncopharmacology Unit, Centre Antoine Lacassagne, 33 Avenue de Valombrose, Nice, Cedex 2, 06189, France; Pharmacology Unit, Centre Alexis Vautrin, Avenue de Bourgogne, Vandoeuvre-les-Nancy Cedex, 54511, France; Pharmacokinetics Unit, Centre René Gauducheau, Bd Jacques Monod, Saint-Herblain Cedex, 44805, France

**Keywords:** 5-fluorouracil, dihydropyrimidine dehydrogenase, anti-cancer drug-related toxicity

## Abstract

Dihydropyrimidine dehydrogenase (DPD) is the initial and rate-limiting enzyme of 5-fluorouracil (5-FU) catabolism. We report lymphocytic DPD data concerning a group of 53 patients (23 men, 30 women, mean age 58, range 36–73), treated by 5-FU-based chemotherapy in different French institutions and who developed unanticipated 5-FU-related toxicity. Lymphocyte samples (standard collection procedure) were sent to us for DPD determination (biochemical method). Among the whole group of 53 patients, 19 had a significant DPD deficiency (DD; below 150 fmol min^−1^ mg^−1^ protein, i.e. less than 70% of the mean value observed from previous population study). There was a greater majority of women in the DD group (15 out of 19, 79%) compared with the remaining 34 patients (15 out of 34, 44%, *P*<0.014). Toxicity was often severe, leading to patient death in two cases (both women). The toxicity score (sum of WHO grading, theoritical range 0–20) was twice as high in patients with marked DD (below 100 pmol min^−1^ mg^−1^ protein, *n* = 11, mean score = 13.2) compared with patients with moderate DD (between 150 and 100 pmol min^−1^ mg^−1^ protein, *n* = 8, mean score = 6.8), *P* = 0.008. In the DD group, there was a high frequency of neurotoxic syndromes (7 out of 19, 37%). The two deceased patients both had severe neurotoxicity. The occurrence of cardiac toxicity was relatively rare (1 out of 19, 5%). These data suggest that women are particularly prone to DPD deficiency and allow a more precise definition of the DD toxicity profile. © 1999 Cancer Research Campaign


*British Journal of Cancer* (1999) **79**(3/4), 627–630
© 1999 Cancer Research Campaign
Article no. bjoc.1998.0098

### Dihydropyrimidine dehydrogenase deficiency and fluorouracil-related toxicity

**G Milano, MC Etienne, V Pierrefite[1], M Barberi-Heyob[2], R Deporte-Fety[3] and N Renée**

[1]Centre Antoine Lacassagne, Oncopharmacology Unit, 33, Avenue de Valombrose, 06189 Nice Cedex 2; [2]Centre Alexis Vautrin, Pharmacology Unit, Avenue de Bourgogne, 54511 Vandoeuvre-les-Nancy Cedex; [3]Centre René Gauducheau, Pharmacokinetics Unit, Bd Jacques Monod, 44805 Saint-Herblain Cedex, France

**Summary** Dihydropyrimidine dehydrogenase (DPD) is the initial and rate-limiting enzyme of 5-fluorouracil (5-FU) catabolism. We report lymphocytic DPD data concerning a group of 53 patients (23 men, 30 women, mean age 58, range 36–73), treated by 5-FU-based chemotherapy in different French institutions and who developed unanticipated 5-FU-related toxicity. Lymphocyte samples (standard collection procedure) were sent to us for DPD determination (biochemical method). Among the whole group of 53 patients, 19 had a significant DPD deficiency (DD; below 150 fmol $min^{-1}$ $mg^{-1}$ protein, i.e. less than 70% of the mean value observed from previous population study). There was a greater majority of women in the DD group (15 out of 19, 79%) compared with the remaining 34 patients (15 out of 34, 44%, $P$<0.014). Toxicity was often severe, leading to patient death in two cases (both women). The toxicity score (sum of WHO grading, theoritical range 0–20) was twice as high in patients with marked DD (below 100 pmol $min^{-1}$ $mg^{-1}$ protein, $n$ = 11, mean score = 13.2) compared with patients with moderate DD (between 150 and 100 pmol $min^{-1}$ $mg^{-1}$ protein, $n$ = 8, mean score = 6.8), $P$ = 0.008. In the DD group, there was a high frequency of neurotoxic syndromes (7 out of 19, 37%). The two deceased patients both had severe neurotoxicity. The occurrence of cardiac toxicity was relatively rare (1 out of 19, 5%). These data suggest that women are particularly prone to DPD deficiency and allow a more precise definition of the DD toxicity profile.

**Keywords:** 5-fluorouracil; dihydropyrimidine dehydrogenase; anti-cancer drug-related toxicity

More than 80% of an administered dose of fluorouracil (FU) is eliminated by catabolism through dihydropyrimidine dehydrogenase (DPD), the rate-limiting enzyme of pyrimidines (Diasio and Harris, 1989). Tuchman and colleagues (1985) were the first to describe a patient with severe FU toxicity associated with a pyrimidine metabolism disorder. On the basis of another case report, Diasio et al (1988) conducted a familial study, the conclusions of which suggested an autosomal recessive pattern of inheritance for DPD deficiency. Since then, similar pharmacogenetic syndromes have been reported by the same group (Harris et al, 1991; Lu et al, 1993), others (Houyau et al, 1993) and ourselves (Fleming et al, 1993). We recently performed a review analysis on previously published cases of DPD deficiency associated with FU toxicity. It appeared that a complete absence of DPD activity is extremely rare and even partial enzyme activity might result in more or less severe FU toxicity (Milano and Etienne, 1994). In addition, the fact that among 15 cumulated cases (most with digestive cancer) 13 (87%) were women was very striking and suggestive of a sex-linked deficiency in DPD activity. We report on the clinical and pharmacological results from 19 cancer patients with more or less intense FU-related toxicity, who were phenotyped in our centre as carrying a DPD deficiency diagnosed in peripheral blood mononuclear cells. Different features were analysed including sex ratio, the toxicity profile, and a possible link between the intensity of DPD deficiency and the severity of FU toxicity.

*Received 16 January 1998*
*Revised 30 April 1998*
*Accepted 9 May 1998*

*Correspondence to:* G Milano

### MATERIAL AND METHODS

### Patients

This study represents a 3-year collection of blood lymphocytes taken from 53 consecutive patients treated by FU-based chemotherapy in different French institutions (general hospitals, cancer centres, private hospitals). These patients had experienced unpredicted more or less severe FU-related toxicity. Blood lymphocytes were collected in the respective hospitals within a period of 1 month after the FU toxicity episode. All lymphocyte samples were shipped in dry-ice to our laboratory. Among this group of 53 patients (23 men, 30 women, mean age 58, range 36–73), 19 exhibited a moderate or marked DPD deficiency (less than 70% of the mean population value, i.e. less than 150 fmol $min^{-1}$ $mg^{-1}$ protein; Etienne et al, 1994). A complete description of these 19 case reports is given in Table 1. We defined a toxicity score which was the sum of the toxicity grades (WHO classification) for mucositis, neutropenia, thrombocytopenia and digestive toxicity. The presence of neurotoxicity was graded 4. The maximal toxicity score was 20.

### Determination of DPD activity

Lymphocyte preparation was carried out in the hospital where the patient received the FU-based treatment. Because of a known circadian pattern of DPD activity (Harris et al, 1990), blood samples were drawn between 8 a.m. and 10 a.m. to minimize the influence of circadian variability. When patients developed severe haematological toxicity, a normalization of blood cell count (white blood cells) was awaited before collecting blood samples for DPD determination. The lymphocytes were prepared as previously described (Fleming et al, 1993). All the lymphocyte pellets were brought to our centre, where they were kept at –80°C, and DPD determination was performed within 2 weeks. DPD activity was determined as previously described (Fleming et al, 1993). Briefly, the assay involved incubating lymphocyte extract (cytosol) with [$^{14}$C]FU, followed by high-performance liquid chromatography (HPLC) separation and quantification of [$^{14}$C]$FUH_2$. Enzyme activity was expressed as fmol of $FUH_2$ formed per minute and per milligram of protein. The sensibility limit was 10 pmol $min^{-1}$ $mg^{-1}$ protein. The interassay reproducibility (aliquots of a pooled lymphocyte suspension) gave a coefficient of variation of 12%.

### Statistics

Comparisons of frequencies were carried out by chi-squared test. Group comparisons were carried out by the Mann–Whitney test. Statistics were drawn up on SPSS software (Chicago, IL, USA).

### RESULTS

Table 1 summarizes the clinical, pharmacological and biological data concerning the 19 study patients with unanticipated FU-related toxicity and significant DPD deficiency (lymphocytic DPD activity below 150 pmol $min^{-1}$ $mg^{-1}$ protein). Most FU chemotherapy protocols included FU modulation by folinic acid (12 out of 19, 63%). In four cases, patients had previously received a FU-based chemotherapy (more than 1 year before the present treatment). In two of them, patients 14 and 15, it was possible to retrieve an indication of FU-related toxicity (mild neurotoxicity for patient 14 and haematological toxicity for patient 15). There was a marked majority of women in this group of DPD deficient patients (15 out of 19, 79%). This population of women was significantly higher ($P$ = 0.014) than that found in the remaining 34 patients (15 women out of 34, 44%), whose DPD was higher than 150 pmol $min^{-1}$ $mg^{-1}$ protein. Toxicities were often severe, leading to patient death in two cases (both women). The toxicity profile was typically related to FU in most cases (mucositis, neutropenia). The toxicity score was significantly higher in patients with markedly low DPD (below 100 pmol $min^{-1}$ $mg^{-1}$ protein) compared with patients with moderate DPD deficiency (below 150 and above or equal to 100 pmol $min^{-1}$ $mg^{-1}$ protein); the mean values of the toxicity score were 13.2 ± 5.9 ($n$ = 11) and 6.8 ± 3.7 ($n$ = 8) respectively ($P$ = 0.008). There was a high proportion of neurotoxic syndromes (7 out of 19, 37%). The two deceased patients both had neurotoxicity. In contrast, the occurrence of cardiac toxicity was relatively rare (1 out of 19, 5%).

### DISCUSSION

The present study provides clinical, pharmacological and biological data concerning 19 new cases of FU toxicity in relation to DPD deficiency. The group of patients analysed in the study represents more than the total number of isolated case reports published so far in the literature. The notion of DPD deficiency is hard to delineate precisely because it has been dependent upon the determination of lymphocytic DPD activity performed by different laboratories and, thus, included some degree of unavoidable analytical variability. The present prospective investigation performed in a single site had the advantage of avoiding this predictable interinstitution variability in DPD determination. After the analysis of lymphocyte samples taken from 53 patients who had developed an unexpected, more or less severe, FU-related toxicity, 19 (36%) revealed a relative DPD deficiency (below 150 pmol $min^{-1}$ $mg^{-1}$ protein, i.e. less than 70% of the mean population value; Etienne et al, 1994). Similarly, Lu et al (1993) reported that in 25 cancer patients who had experienced moderate to severe FU-related toxicity, nine (36%) were found to have demonstrated a more or less marked DPD deficiency at lymphocyte level. It is clear that causes other than DPD deficiency can predispose to FU toxicity; these causes may include impaired liver function (Floyd et al, 1982) and altered nutritional status (Stenram, 1993). It was not within the scope of the present study to investigate thoroughly the diverse origins of the FU-attributed toxic manifestations after FU-based chemotherapy in the whole group of patients.

One of the main findings which emerged from the exploration of the study patients is that the great majority of cases are women (79%). This observation cannot be explained by a sex-related tumour profile because most cases were digestive tract cancers. The proportion of women in the study group was significantly higher than in the remaining 34 patients from the whole group (15 women out of 34, i.e. 44%), whose DPD activity was not altered. This observation confirms the conclusions of earlier analyses in previously published case reports in which it was shown that women constituted 87% of DPD-deficient patients with FU-related toxicity (Milano and Etienne, 1994). We recently performed a prospective study on a large group of 185 unselected cancer patients (Etienne et al, 1994) and showed that lymphocytic DPD activity was, on average, 15% lower in women than in men. Interestingly, this 15% difference in DPD activity was of the order of magnitude as that observed for FU clearance between men and women (Milano et al, 1992). In contrast, in a population study by Lu et al (1993), DPD activity was not influenced by sex. It must be stressed that the *DPD* gene is not carried by a sexual chromosome, but by chromosome 1 (Yokota et al, 1994). Thus, the cause of the high frequency of women in DPD deficient patients is rather difficult to explain. The present data show that female patients are prone to carry a DPD-deficiency syndrome and should be given priority in systematic investigations of DPD activity before FU-based chemotherapy. This approach does not preclude careful consideration of the cost–benefit ratio.

Neurotoxicity is rarely reported among the undesirable side-effects of FU chemotherapy (Langer et al, 1996). Takimoto and colleagues (1996) recently reported on a patient with DPD deficiency who developed severe neurotoxicity after 5FU-based chemotherapy. The present study clearly indicates that neurotoxicity plays a significant part in the toxicity profile exhibited by DPD-deficient patients after FU treatment. Neurotoxicity was predominant when DPD deficiency was marked: among the 11 patients with lymphocytic DPD below 100 fmol $min^{-1}$ $mg^{-1}$ protein, seven (including two toxic deaths) developed more or less severe toxicity. The aetiology of FU-related neurotoxicity is still poorly understood. In agreement with a previous report (Diasio et al, 1988), it is suggested that neurotoxicity could be attributable to marked and prolonged exposure to FU in the CSF, secondary to plasma FU overexposure due to impaired drug clearance related to the DPD deficiency.

In contrast to neurotoxicity, patients with DPD deficiency did not present a particularly high incidence of cardiotoxicity because only one case (5%) was observed in the present study. The mechanisms of FU cardiotoxicity are unknown, but it is probable that this form of toxicity cannot be accounted for by FU pharmacokinetic

© Cancer Research Campaign 1999

**Table 1** Patients with DPD deficiency

| Patient no. (sex, age) | Primary cancer | FU-based treatment | DPD | | FU-related toxicity | | | | | |
|---|---|---|---|---|---|---|---|---|---|---|
| | | | Lymphocytic activity[a] | Δ t (weeks) | Mucositis (grade) | Neutropenia (grade) | Thrombo-cytopenia (grade) | Digestive toxicity (grade) | Neurological toxicity | Others |
| 1 (F, 66) | P | FU bolus 400 mg m$^{-2}$ × 5 + adriblastin (cycle 1) | 143 | 4 | 4 | 4 | 4 | 2 (diarrhoea) | No | Septicaemia |
| 2 (F, 47) | C | FUFOL (3 h) 600 mg m$^{-2}$ day$^{-1}$ × 5 (cycle 1) | 146 | 4 | 2 | 4 | No | No | No | Alopecia grade 3 |
| 3 (F, 60) | C | FUFOL bolus 375 mg m$^{-2}$ day$^{-1}$ × 5 (cycle 2) | 101 | 2 | No | 2 | No | No | No | Cardiotoxicity alopecia grade 3 |
| 4 (F, 44) | C | FU bolus 675 mg m$^{-2}$ day$^{-1}$ × 5 + levamisole (cycle 1) | 61,40 | 12 | 4 | 3 | 3 | | | |
| 5 (M, 58) | C | FUFOL (cycle 1) | 107 | 4 | No | No | No | 3 (diarrhoea) | No | Septicaemia |
| 6 (F, 56) | C | FUFOL (cycle 1) | 65 | 8 | 2 | No | No | 2 | No | |
| 7 (F, 61) | B | FU continuous infusion (cycle 1) | 34 | 16 | 2 | No | 2 | No | No | |
| 8 (F, 48) | C | FU (48 h) 105 mg m$^{-2}$ day$^{-1}$ (cycle 1) | 122 | 4 | 3 | 4 | No | 1 | No | |
| 9 (F, 45) | B | FU bolus 900 mg day$^{-1}$ + novantrone + cytoxin (cycle 1) | 85 | 4 | 4 | 3–4 | 3–4 | 4 (diarrhoea) | Yes (stupor) | |
| 10 (M, 60) | C | FUFOL 750 mg m$^{-2}$ day$^{-1}$ (cycle 4) | 14 | 4 | 4 | 4 | No | 4 | No | |
| 11 (M, 66) | O | FU continuous infusion 1 g m$^{-2}$ day$^{-1}$ × 5 + cisplatin (cycle 1) pretreated | 88 | 6 | 3 | 4 | 4 | 3 (diarrhoea) | Yes (cerebellar syndrome) | |
| 12 (F, 73) | R | FUFOL continuous infusion 400 mg m$^{-2}$ day$^{-1}$ × 5 (cycle 1) | 100 | 4 | 3 | 3 | No | No | No | Cutaneous toxicity |
| 13 (F, 63) | R | FUFOL Oxaliplatin (cycle 1) pretreated | 122 | 3 | No | No | No | 4 (diarrhoea) | Yes (drowsiness confusion) | |
| 14 (F, 51) | B | FU Navelbine (cycle 1) pretreated | 61 | 2 | 4 | 4 | 3 | 4 (diarrhoea) | Yes (comatose state) | Toxic death |
| 15 (F, 64) | C | FUFOL 625 mg m$^{-2}$ day$^{-1}$ × 5 (cycle 1) pretreated | 48 | 3 | 4 | 4 | 4 | No | Yes (confusion) | Fever |
| 16 (F, 36) | C | FUFOL (cycle 1) | 87 | 2 | 4 | No | No | 2 (diarrhoea) | No | |
| 17 (F, 52) | C | FUFOL continuous 375 mg m$^{-2}$ day$^{-1}$ × 5 (cycle 1) | 135 | 4 | 4 | 4 | No | No | No | Alopecia grade 3 |
| 18 (M, 68) | C | FUFOL continuous 425 mg m$^{-2}$ day$^{-1}$ (cycle 1) | 53 | 2 | 4 | 4 | 4 | No | Yes (visual troubles) | |
| 19 (F, 61) | P | FU cisplatin continuous 1 g m$^{-2}$ day$^{-1}$ × 5 (cycle 1) | 10 | 3 | 4 | 4 | 4 | No | Yes (comatose state) | Toxic death |

F, female; M, male; Primary cancer: P, pancreas; C, colon; O, oesophagus; B, breast; R rectum; FU-based treatment: pretreated means pretreated with FU containing chemotherapy; FUFOL means FU + folinic acid-based chemotherapy; Δ t means the time interval between the toxic cycle and blood sampling for DPD determination; [a]pmol min$^{-1}$ mg$^{-1}$ protein.

*© Cancer Research Campaign 1999*

abnormalities with drug overexposure due to DPD deficiency. This view strengthens previous findings by our group in which FU pharmacokinetic-monitored patients who developed FU-related cardiotoxicity did not present abnormal FU plasma concentrations (Thyss et al, 1988).

Another aspect of the present study is the fact that none of the reported cases had a complete DPD deficiency, even considering the two female patients who died from severe DPD toxicity. Patients with moderate DPD deficiency (between 100 and 150 fmol $min^{-1}$ $mg^{-1}$ protein) were predisposed to more or less severe FU-related toxicity. The severity of the toxicity was shown, however, to match the intensity of the DPD deficiency. This can be explained by a more or less marked overexposure to FU linked to the intensity of DPD deficiency because a significant relationship between lymphocytic DPD activity and FU clearance has been previously reported (Fleming, 1992).

A molecular basis for DPD deficiency was recently suggested (Wei et al, 1996). The authors found that a G to A mutation within the 5′ splicing site appeared to cause exon skipping, leading to an inactive *DPYD* allele. The physical map of the *DPD* gene was published recently (Johnson et al, 1997). The classical assay for DPD activity determinations, as used in the present paper, is labour intensive and is not to be applied on a large scale. The recent basic findings concerning DPD should permit the development of rapid assays to detect mutations in the *DPD* gene. But it is not certain that genotyping will constitute a better solution than the current phenotyping approach, as different subjects genotyped as heterozygous for the 5′ splicing mutation may exhibit highly variable lymphocytic DPD activity among themselves (Wei et al, 1996). Further studies need to be conducted with both genotyping and phenotyping to assess DPD deficiency and the risk of FU-related toxicity.

### REFERENCES

Diasio RB and Harris BE (1989) Clinical pharmacology of 5-fluorouracil. *Clin Pharmacokinet* **16**: 215–237

Diasio RB, Beavers TL and Carpenter JT (1988) Familial deficiency of dihydropyrimidine dehydrogenase. Biochemical basis for familial pyrimidinemia and severe 5-fluorouracil induced toxicity. *J Clin Invest* **81**: 47–51

Etienne MC, Lagrange JL, Dassonville O, Fleming R, Thyss A, Renée N, Schneider M, Demard F and Milano G (1994) Population study of dihydropyrimidine dehydrogenase in cancer patients. *J Clin Oncol* **12**: 2248–2253

Fleming R, Milano G, Thyss A, Etienne MC, Renée N, Schneider M and Demard F (1992) Correlation between dihydropyrimidine dehydrogenase activity in peripheral mononuclear cells and systemic clearance of fluorouracil in cancer patients. *Cancer Res* **52**: 2899–2902

Fleming RA, Milano G, Gaspard MH, Bargnoux PJ, Thyss A, Plagne R, Renée N, Schneider M and Demard F (1993) Dihydropyrimidine dehydrogenase activity in cancer patients. *Eur J Cancer* **29**: 740–744

Floyd RA, Hornbeck CL and Byfield JE et al (1982) Clearance of continuously infused 5-fluorouracil in adults having lung or gastrointestinal carcinoma with or without hepatic metastases. *Drug Intell Clin Pharm* **16**: 665–670

Harris BE, Song R, Soong SJ and Diasio RB (1990) Relationship between dihydropyrimidine dehydrogenase activity and plasma 5-fluorouracil levels with evidence for circadian variation of enzyme activity and plasma drug levels in cancer patients receiving 5-fluorouracil by protracted continuous infusion. *Cancer Res* **50**: 197–201

Harris BE, Carpenter JT and Diasio RB (1991) Severe 5-fluorouracil toxicity secondary to dihydropyrimidine dehydrogenase deficiency. A potentially more common pharmocogenetic syndrome. *Cancer* **50**: 197–201

Houyau P, Gay C, Chatelut E, Canal P, Roche H and Milano G (1993) Severe flourouracil toxicity in a patient with dihydropyrimidine dehydrogenase deficiency. *J Natl Cancer Inst* **85**: 1602–1603

Johnston MR, Weang K, Tillmans S, Albin N and Diasio RB (1997) Structural organization of the human dihydropyrimidine dehydrogenase gene. *Cancer Res* **57**: 1660–1663

Langer CJ, Hageboutros A, Kloth DD, Roby D and Shaer AH (1996) Acute encephalopathy attributed to 5-FU. *Pharmacotherapy* **16**: 311–313

Lu Z, Zhang R and Diasio RB (1993) Dihydropyrimidine dehydrogenase activity in human peripheral blood mononuclear cells and liver: population and clinical implication in 5-fluorouracil chemotherapy. *Cancer Res* **53**: 5433–5438

Milano G and Etienne MC (1994) Potential importance of dihydropyrimidine dehydrogenase in cancer chemotherapy. *Pharmacogenetics* **4**: 301–306

Milano G, Etienne MC, Thyss A, Santini J, Frenay M, Renée N, Schneider M and Demard F (1992) Influence of sex and age on fluorouracil clearance. *J Clin Oncol* **10**: 1171–1175

Stenram U (1993) 5-FU toxicity and nutritional deficiencies. *Br J Cancer* **67**: 1157

Takimoto CH, Lu ZH, Zhang R, Liang MD, Larson LV, Cantilena LR, Grem JL, Allegra CJ, Diasio RB and Chu E (1996) Severe neurotoxicity following 5-fluorouracil-based chemotherapy in a patient with dihydropyrimidine dehydrogenase deficiency. *Clin Cancer Res* **2**: 477–481

Thyss A, Milano G, Schneider M and Demard F (1988) Circulating drug levels in patients presenting cardiotoxicity to 5-FU. *Eur J Cancer Clin Oncol* **24**: 1675–1676

Tuchman M, Stoekeler JS, Kiang DT, O'Dea R, Ramnaraine ML and Mirkia BL (1985) Familial pyrimidinemia and pyrimidinuria associated with severe fluorouracil toxicity. *N Engl J Med* **313**: 245–249

Wei X, McLeod HL, McMurrough J, Gonzalez FJ and Fernandez-Salguero P (1996) Molecular basis of the human dihydropyrimidine dehydrogenase deficiency and 5-fluorouracil toxicity. *J Clin Invest* **98**: 610–615

Yokota H, Fernandez-Salguero P, Furuya H, Lin K, McBride OW, Podschum B, Schnackerz KD and Gonzalez FJ (1994) cDNA cloning and chromosome mapping of human dihydropyrimidine dehydrogenase, an enzyme associated with 5-fluorouracil toxicity and congenital thymine uraciluria. *J Biol Chem* **269**: 23192–23196

© Cancer Research Campaign 1999
